# Exploring psychological preparedness for volcanic hazards in early childhood education: A sequential explanatory mixed-methods approach in Ternate Island

**DOI:** 10.4102/jamba.v18i1.2127

**Published:** 2026-08-14

**Authors:** Dewi M. Ummah, Koentjoro Soeparno, Muh Aris Marfai, Pradytia P. Pertiwi

**Affiliations:** 1 Department of Psychology, Faculty of Psychology, Gadjah Mada University, Yogyakarta, Indonesia; 2 Department of Geography, Faculty of Geography, Gadjah Mada University, Yogyakarta, Indonesia

**Keywords:** early childhood education, psychological preparedness, *tripusat pendidikan*, volcanic hazards, small-island communities

## Abstract

Small volcanic islands are characterised by high hazard exposure and structural vulnerability, conditions that may increase psychological risks for young children. Although disaster preparedness has received increasing attention in early childhood education (ECE), psychological preparedness in volcanic small-island settings remains underexplored. This mixed-methods study examined psychological preparedness among ECE stakeholders on Ternate Island, Indonesia, using survey data from 127 participants and six focus group discussions within the *tripusat pendidikan* framework of family, school and community. Participants demonstrated moderate to high levels of psychological preparedness across stakeholder groups. Within-individual factors (WIF) showed the highest mean score (*M* = 22.94, s.d. = 5.69), followed by individual-institutional factors (IIF) (*M* = 6.46, s.d.= 1.94) and between-individual factors (BIF) (*M* = 1.89, s.d. = 1.07). No significant differences were found among teachers, parents and community members (*p* > 0.05). Qualitative findings revealed culturally embedded coping resources, including self-efficacy, religious meaning-making and collective coping, alongside limited eruption-specific knowledge, weak emotional regulation strategies and inconsistent communication across relational and institutional levels. Integrated findings indicate an ecological asymmetry in preparedness, whereby strong individual resilience coexists with weaker relational and institutional coordination. Psychological preparedness appears to depend more on cultural coping systems and shared community experiences than on formal disaster governance.

**Contribution:**

These findings highlight the need to strengthen coordination across the *tripusat pendidikan* ecosystem to foster sustainable and child-centred psychological preparedness in volcanic small-island communities.

## Introduction

Ternate Island in North Maluku, Indonesia, is a small active volcanic island with an extremely high level of disaster risk. Existing studies have noted that the combination of threats from the active volcano and local structural vulnerability is the core risk-driving factor (Hidayat, Marfai & Hadmoko 2020; Hidayat et al. 2023). The island also holds multiple pressures related to its geomorphology and social development. Approximately 63% of the population resides in high-risk zones, leading to an extremely severe level of vulnerability (Andreastuti et al. 2023; Barclay et al. 2019; BNPB 2019; Hidayat et al. 2020, 2023). The island’s geographic isolation further limits access to external assistance and emergency resources (Abenir et al. 2022; Hidayat, Marfai & Hadmoko 2019). These conditions increase social, economic and psychological risks, particularly among children who are vulnerable to fear, anxiety and post-disaster stress (La Greca et al. 2013; Lai & La Greca 2020; Lai et al. 2020; Peek 2008). The map here depicts the location of this study.

Psychological preparedness in disaster settings is reflected in how individuals manage emotional, cognitive and social responses when anticipating, facing and recovering from threatening events (Donahue, Eckel & Wilson 2014; Paton 2019). Unlike physical preparedness, psychological preparedness relates more closely to how individuals regulate emotions, maintain a sense of control and cope with stress and uncertainty during threatening situations (Boylan & Lawrence 2020; Clode 2010; Every et al. 2019; Paton 2019). These capacities are especially important in early childhood, a developmental period in which emotional regulation and coping abilities are still emerging, making children more vulnerable to psychological distress during disasters (Estafetta et al. 2020; Guterman 2005; Zulch 2019). In the Indonesian context, psychological preparedness has been conceptualised through the PREPARED framework, which includes within-individual factors (WIF), between-individual factors (BIF) and individual-institutional factors (IIF). In Indonesia, psychological preparedness has been conceptualised through the PREPARED framework, which describes preparedness across three interconnected dimensions: WIF, BIF and IIF (Pertiwi et al. 2023, 2026).

Young children rarely develop psychological preparedness independently because their understanding of danger, emotional regulation and sense of safety are shaped through ongoing interactions with significant adults, including parents and early childhood educators (Amri et al. 2018; Masten 2010; Peek 2008; Ronan et al. 2015). In disaster situations, adults play an important role in helping children interpret threatening events, regulate emotions and maintain a sense of safety (Amri et al. 2018). Within early childhood education (ECE) settings, teachers and caregivers also become sources of emotional reassurance and disaster-related learning that support children’s adaptive responses through observation, routine interactions and guided experiences (Blum et al. 2008; Paton 2013; Pfefferbaum, Pfefferbaum & Van Horn 2018). This relational perspective aligns closely with Ki Hadjar Dewantara’s concept of *tripusat pendidikan*, which highlights the interconnected roles of family, school and community in child development (Ki Hadjar Dewantara 2013). From a socio-ecological perspective, families contribute to emotional security and early risk awareness, schools support disaster learning and emotional guidance, while communities provide broader social and institutional support systems (Boon et al. 2012; Paton 2013). Similar ideas can also be found in Bronfenbrenner’s ecological systems theory and disaster risk reduction (DRR) frameworks that emphasise cross-sector collaboration in strengthening children’s preparedness and resilience (Boon et al. 2012; Bouchard & Smith 2017; Pfefferbaum et al. 2012).

Although research on disaster preparedness in early childhood settings has expanded in recent years, studies examining psychological preparedness in volcanic small-island contexts remain limited. Existing literature has focused primarily on physical preparedness, with less attention given to psychological dimensions and the interconnected roles of family, school and community within socio-ecological systems. Furthermore, there is a relative scarcity of mixed-methods studies that investigate preparedness within culturally grounded disaster contexts. To address this gap, the present study examines psychological preparedness among ECE stakeholders on Ternate Island, Indonesia, through the *tripusat pendidikan* framework. The study further explores how relational, cultural and institutional factors shape preparedness within a volcanic small-island setting while providing practical insights for more child-centred DRR in vulnerable island communities.

## Research methods and design

### Study design

A sequential explanatory mixed-methods design was employed, consisting of a quantitative phase followed by a qualitative phase to further explain and contextualise the findings (Creswell 2018). The validated 41-item PREPARED tool was used to assess psychological preparedness across three dimensions: WIF, BIF and IIF (Pertiwi et al. 2023, 2026). The questionnaire was administered in both online and printed formats to accommodate limited digital literacy and demonstrated strong internal consistency (Cronbach’s α = 0.916). In the qualitative phase, focus group discussions (FGDs) were conducted to explore participants’ experiences and provide a deeper interpretation of the quantitative results. Findings from both phases were integrated through triangulation to enhance interpretive validity (Fetters & Tajima 2022; Guetterman, Fetters & Creswell 2015). The procedural flow of the study is presented in Figure 2.

**FIGURE 1:**
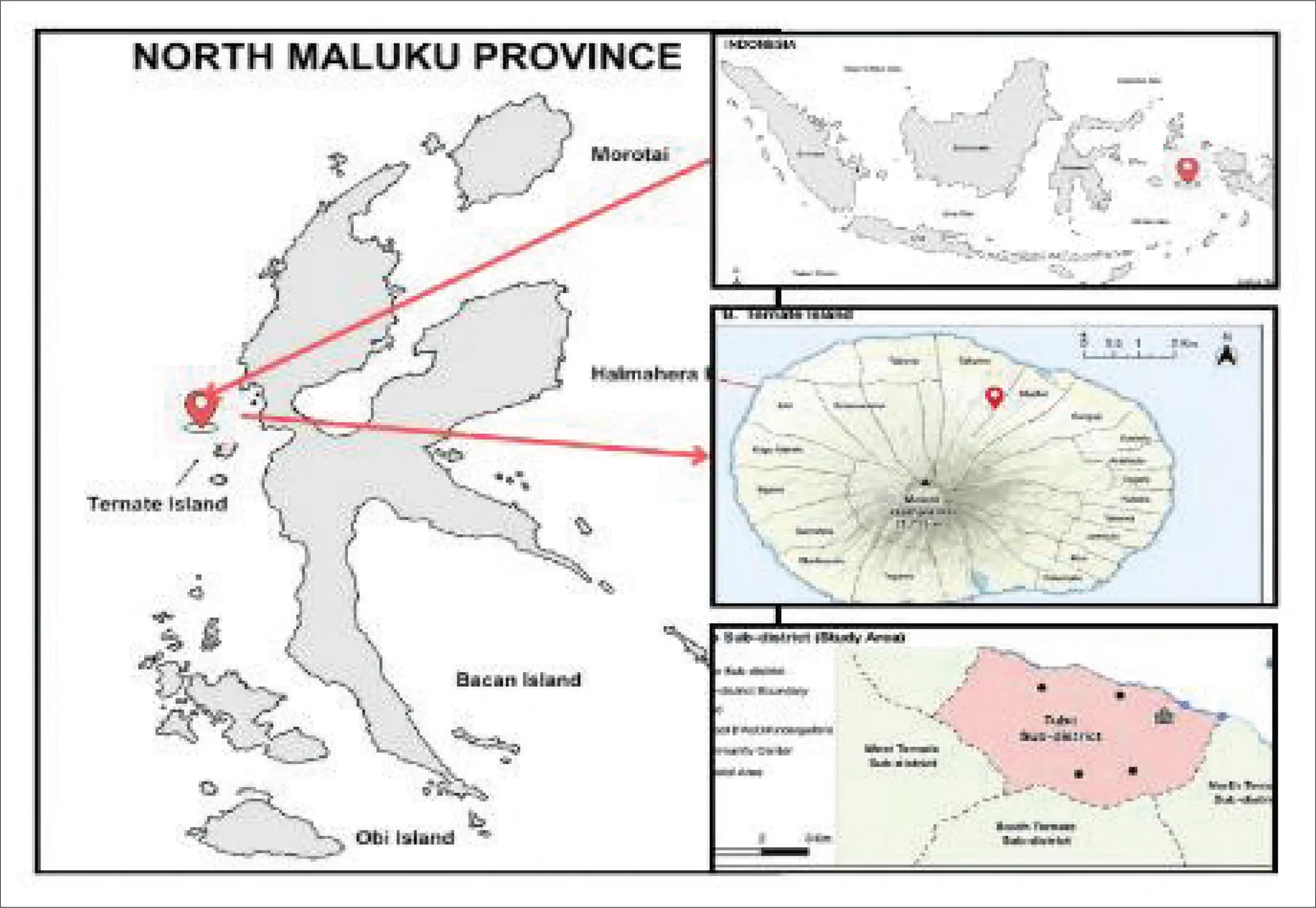
Study location Tubo sub-district, Ternate Island, North Maluku Province, Indonesia.

**FIGURE 2:**
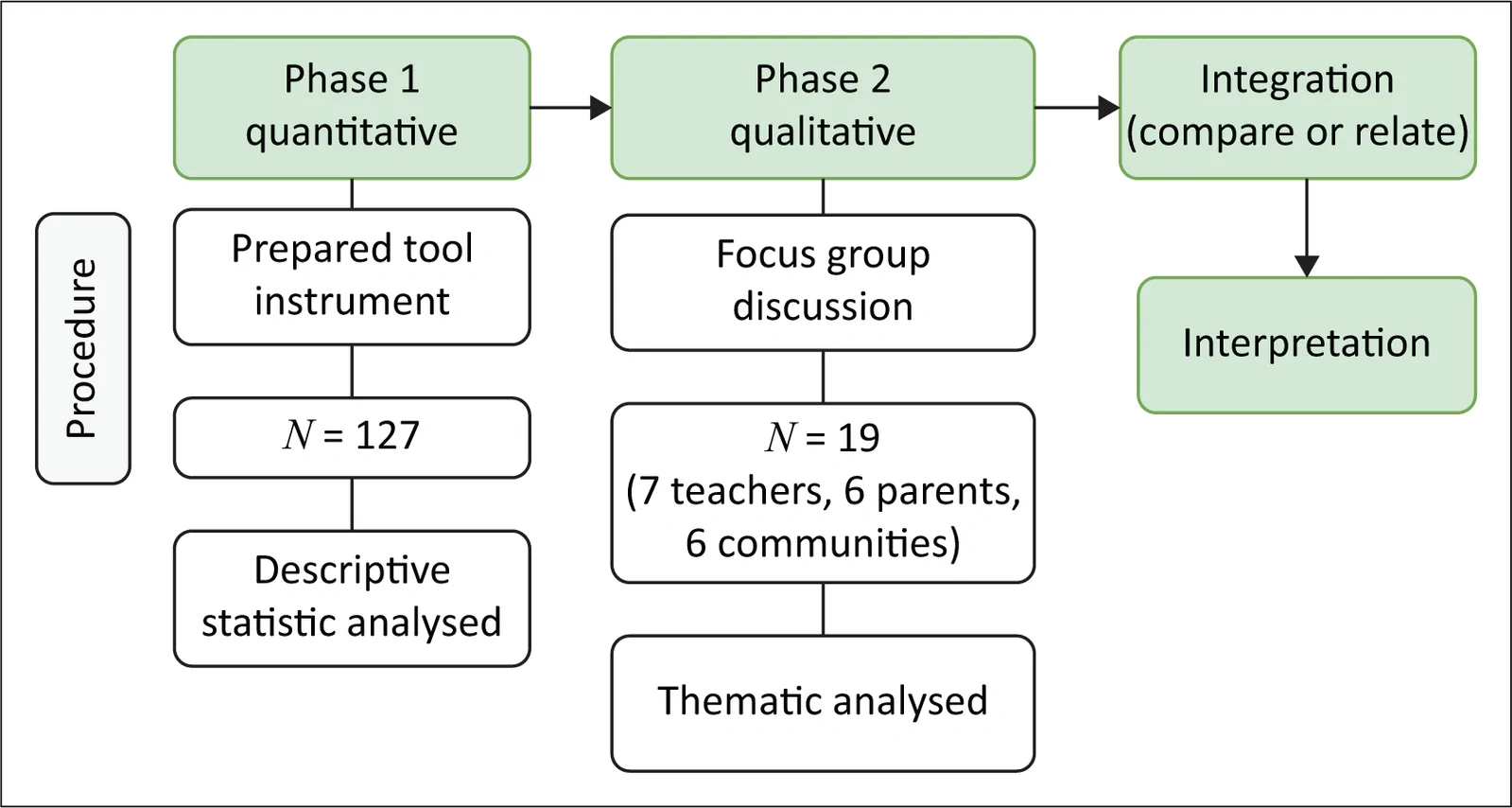
Procedural diagram of research.

**FIGURE 3:**
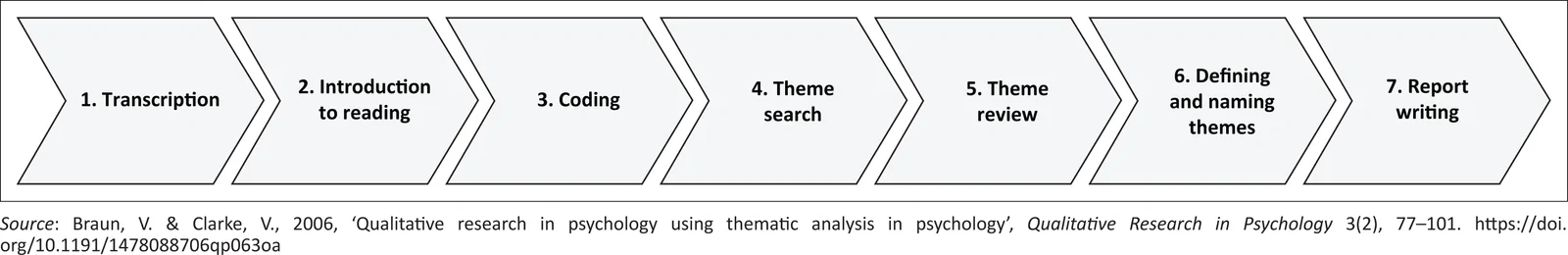
Stages of qualitative data analysis.

### Participants and sampling

Purposive sampling was used to recruit adults involved in early childhood caregiving or education, including parents, teachers and community members, residing in Tubo sub-district, a Mount Gamalama hazard-prone area (Marfai, Mei & Retnowati 2019; Taherdoost 2018). Inclusion criteria included being aged 18 years or older, residing in the eruption-prone area, and having at least a high school education. Participants who were unwilling or unable to complete the questionnaire were excluded.

Quantitative participants were recruited offline through face-to-face questionnaire distribution conducted in collaboration with Khairun University. Recruitment was carried out through community networks and ECE settings in eruption-prone areas of Ternate Island. No randomisation was applied, as the study focused on participants with direct experience living in volcanic hazard areas.

For the qualitative phase, participants with low to moderate PREPARED scores were invited to join FGDs to further explain the quantitative findings. Six FGDs were conducted with teachers (*n* = 7), parents (*n* = 6) and community members (*n* = 6), each lasting approximately 75–90 min.

### Data collection

Participants were contacted through WhatsApp and direct visits within the community. Before data collection began, all participants received information about the study, provided written informed consent and completed a demographic form together with the PREPARED tool, an instrument designed to assess psychological preparedness across 15 constructs within three domains. Preparedness scores were then grouped into five categories ranging from very low to very high. Focus group discussions were carried out face-to-face using a semi-structured interview guide adapted from the PREPARED framework. Prior to the main data collection, the guide was piloted with five individuals to ensure clarity and relevance. With participants’ permission, all discussions were audio-recorded and conducted at mutually agreed times and locations (Pertiwi et al. 2023).

### Data analysis

Quantitative data were analysed using SPSS version IBM 27. Descriptive statistics (means, standard deviations and percentages) were used to summarise demographic characteristics, preparedness levels and group differences.

Qualitative data were analysed using thematic analysis following Braun and Clarke (2006). Audio recordings were transcribed verbatim. Two researchers independently coded the transcripts, compared coding results and resolved discrepancies through discussion. Coding continued until data saturation was achieved and no new themes emerged. Identified codes were then organised into patterns and overarching themes reflecting participants’ experiences of psychological preparedness.

Integration of quantitative and qualitative findings was conducted at the interpretation stage to generate meta-inferences explaining preparedness levels within the *tripusat pendidikan* framework featured in a joint display (Fetters & Tajima 2022).

### Ethical considerations

Ethical approval was obtained from the Research Ethics Committee of the Faculty of Psychology, Universitas Gadjah Mada (Ref. No.8649/UNI/FPSi.1.3/SD/PT.01.04/2024). Participation was voluntary, and written informed consent was obtained from all participants. Confidentiality was ensured through anonymisation and the use of pseudonyms. Audio files and transcripts were stored securely with restricted access.

## Results

### Multi-level psychological preparedness

A total of 127 participants were included in the quantitative phase. The majority were female (81%) and had completed high school (61.42%). Most identified as Ternate tribe members (89.92%). In terms of occupation, homemakers comprised the largest group (32.28%), followed by government or state employees (17.32%) and teachers (14.96%). Regarding roles in early childhood caregiving, most participants were parents (70.08%), followed by community members (20.47%) and teachers (9.24%) (Table 1).

**TABLE 1:** Participant demographics (*N* = 127).

Variable	*n*	%
**Gender**		
Female	103	81.00
Male	24	19.00
**Education**		
High school	78	61.42
Bachelor’s or higher	49	38.58
**Occupation**		
Homemaker	41	32.28
Government or state enterprise employee	22	17.32
Teacher	19	14.96
Farmer	16	12.59
Others	29	32.70
**Tribes**		
Ternate	116	89.92
Non ternate	11	8.68
**Early childhood caregivers**		
Parents	89	70.08
Communities	26	20.47
Teacher of early childhood	12	09.24

Quantitative analysis showed that most participants demonstrated moderate to high psychological preparedness (Table 2). Within-individual factors had the highest mean (*M* = 22.94, s.d. = 5.69), followed by IIF (*M* = 6.46, s.d. = 1.94) and BIF (*M* = 1.89, s.d. = 1.07).

**TABLE 2:** Descriptive statistics (*N* = 127).

PREPARED tool domains	Minimum	Maximum	Mean	s.d.
BIF	0	3	1.89	1.071
IIF	1	11	6.46	1.939
WIF	2	30	22.94	5.689

**Total**	**6**	**41**	**31.29**	**7.630**

BIF, Between-individual factors; IIF, Individual–institutional factors; WIF, Within-individual factors; s.d., standard deviation.

Prior to inferential analysis, normality tests were conducted for the PREPARED sub-scale scores using the Kolmogorov-Smirnov test. The results indicated that the distributions of teachers, parents and community member’s scores significantly deviated from normality (*p* < 0.05) (Table 3).

**TABLE 3:** Kolmogorov-Smirnov test.

Statistic	*df*	Sig.
0.135	127	0.000

*df*, degree of freedom; Sig., significance.

Therefore as shown in Table 4, the Kruskal-Wallis test was employed to examine differences in psychological preparedness across stakeholder groups. No significant differences were found among teachers, parents and community members (*p* > 0.05), indicating relatively similar levels of psychological preparedness across ECE stakeholder groups. Shared exposure to volcanic risk and similar socio-cultural experiences may help explain the relatively consistent preparedness perceptions observed among stakeholders within the *tripusat pendidikan* system.

**TABLE 4:** Kruskal-Wallis test.

Kruskal-Wallis H	0.587
*df*	2
Asymp. Sig.	0.746

*df*, degree of freedom; Asymp. Sig., asymptotic significance.

Qualitative findings offered a more nuanced understanding of participants’ psychological preparedness. Many participants described strong personal coping capacities, including awareness of disaster risks, confidence in responding to emergencies and reliance on religious beliefs and everyday coping practices. Although participants were generally familiar with the natural hazards surrounding them, knowledge related to eruption-specific characteristics of Mount Gamalama, such as hazard mapping and spatial risk zones, remained relatively limited. Participants also emphasised the importance of psychological support during eruption situations, particularly in helping children regulate emotions and maintain a sense of safety during times of crisis:

‘Because Tubo is also a disaster-prone area, if the volcano erupts, it will be affected. Even though I don’t know how far Tubo is from the summit of Gamalama, because I’ve never climbed it.’ (Teacher 5, Female, 47 years old)

‘During the panicked situation, I forgot about my child who was still inside the house and fell down the stairs while trying to get out.’ (Parents 2, Female, 48 years old)

Relational and institutional preparedness appeared less consistent across participants. Several respondents described limited communication between schools and families, uneven eruption-specific knowledge among educational stakeholders and irregular institutional involvement in preparedness activities. These conditions may help explain the lower BIF and IIF scores identified in the quantitative findings. As one participant explained, ‘There hasn’t been a place-specific information session here about how to prepare for an eruption’. Overall, the findings highlight an ecological imbalance in which resilience at the microsystem level appeared stronger than coordination across broader mesosystem and exosystem structures.

### Culturally embedded disaster awareness

Within the WIF domain, qualitative findings suggested that participants generally possessed broad knowledge about disasters although this understanding was not always specific to volcanic eruptions. Participants recognised Tubo as a hazard-prone area and were familiar with common volcanic threats. However, more detailed knowledge regarding eruption characteristics, hazard mapping and spatial proximity to Mount Gamalama remained limited. Disaster awareness was also closely tied to religious and cultural perspectives. Participants frequently interpreted disasters through religious narratives and viewed environmental signs, including unusual animal behaviour, as indicators of possible danger. In this context, disaster knowledge appeared to extend beyond technical understanding and included culturally shaped perceptions of risk and environmental change. The integrated findings suggest an important distinction between general disaster awareness and eruption-specific preparedness. While survey results indicated relatively adequate knowledge about disasters, qualitative narratives revealed limitations in more contextual aspects of preparedness, particularly regarding hazard maps, localised risk zones and awareness of spatial proximity to volcanic threats.

### Child-centred preparedness

No statistically significant differences were found across stakeholder groups, suggesting relatively similar patterns of psychological preparedness among teachers, parents and community members. Qualitative findings helped explain this pattern further. Across groups, participants consistently emphasised the importance of supporting children’s emotional well-being during disaster situations, particularly through emotional reassurance, calm behaviour and maintaining daily routines during crises:

‘As one teacher stated, No one was spared from tears, our time a few people. Everyone must embrace the children.’ (Communities 2, Female, 63 years old)

A parent similarly noted:

‘It is essential to minimise the trauma that will affect children in the future when a disaster occurs. So before a disaster occurs, we must prepare for it, discuss it often, and explain it to children so that they begin to understand.’ (Teacher 1, Female, 32 years old)

These narratives suggest that perspectives within the *tripusat pendidikan* ecosystem were shaped more by shared ecological exposure and collective disaster experiences than by differences in professional roles. Participants also acknowledged having limited training in child-focused psychosocial interventions. As a result, approaches to supporting children during disasters appeared to rely largely on intuitive and relational practices rather than on formally institutionalised systems.

### Fragmented institutional coordination

Quantitative findings suggest that institutional preparedness remained relatively modest (IIF: *M* = 6.46, s.d. = 1.94), with Aim C scores lower than those observed at the individual level. Although some formal disaster mitigation frameworks and planning mechanisms were reported, their implementation appeared limited and inconsistent. The absence of significant differences across stakeholder groups also indicates relatively similar perceptions regarding institutional involvement in preparedness activities. Qualitative findings further supported this pattern, as several participants described disaster simulations and collaborations with local authorities as occurring only occasionally:

‘There have been simulations and information campaigns, but only after incidents occurred, and it was a long time ago.’ (Teacher 3, Female, 39 years old)

Others highlighted limited eruption-specific dissemination and irregular school-family communication:

‘We know eruptions can happen, but there is rarely detailed explanation about the map risk zones.’ (Parent 1, Female, 46 years old)

The findings suggest partial convergence between the quantitative and qualitative results. While some institutional mechanisms for disaster preparedness were reported, their implementation appeared irregular and not yet fully systematised. Participants generally demonstrated strong individual coping capacities although coordination across relational and institutional levels remained fragmented. Such conditions may hinder the development of broader collective resilience within the community.

### Integrated meta inference

Table 5 presents the integration of findings through the *tripusat pendidikan* framework, which conceptualises preparedness as a dynamic interplay among *tripusat pendidikan* systems.

**TABLE 5:** Joint display integrating data.

Tripusat-aligned theme	Integrated empirical finding	Meta inference
Individual coping and cultural resilience	Highest WIF; strong self-efficacy and religious coping, but lower in emotional regulation.	*Convergence*. Individual preparedness appeared to be shaped by cultural context and personal confidence although emotional regulation capacities still require strengthening to support adaptive responses during acute stress.
Relational level of family and school	Lowest BIF; informal collaboration; uniform stakeholder responses.	*Complementarity*. There is relational alignment, but it is not officially coordinated. More than position distinction, common exposure shapes shared perspectives.
Level of institutional and community	Moderate IIF; uneven eruption-specific dissemination.	*Convergence*. Institutional structures exist but are not regularly implemented.
Child-centred preparedness	Not directly quantified; emphasis on emotional reassurance and routine stability.	*Expansion*. Child-centred preparedness exists as a relational construct rather than a structural integration.
Cross-domain synthesis	The ecological discrepancies: robust micro-level resilience accompanied by inadequate structural alignment.	*Integration*. Preparedness in volcanic small island contexts illustrates culturally adaptable systems rather than institutional consolidation.
Local cultural knowledge and collective practices	Participants described local cultural expressions such as ‘*bakujaga torang pe ana*’, traditional beliefs to natural signs (*kose)* and collective practices like *kololie kie* as important elements of community-based preparedness.	*Expansion*. Local knowledge and collective cultural practices function as culturally embedded coping systems that support relational and community resilience in volcanic disaster contexts.

BIF, Between-individual factors; IIF, Individual–institutional factors; WIF, Within-individual factors.

Table 5 highlights an ecological imbalance in psychological preparedness across the *tripusat pendidikan* system. Individual resilience appeared relatively strong and closely linked to cultural values and everyday coping practices, while relational coordination and institutional support remained less developed. Overall, preparedness in this volcanic small-island context seemed to rely more on adaptive cultural systems and community-based responses than on formal disaster governance structures.

This arrangement demonstrates that psychologically driven preparedness in small island volcanic environments is maintained primarily through adapted cultural systems rather than through active disaster management. Therefore, enhancing collaboration among components within the *tripusat pendidikan* ecosystem is necessary to transform robust individual coping mechanisms into sustainable, system-wide resilience capable of protecting young children.

## Discussion

This study highlights that preparedness among young children is shaped not only by individual coping capacities but also by relational support systems, cultural values and broader socio-ecological experiences within hazard-prone environments.

### Tripusat Pendidikan as a relational socio-ecological framework for psychological preparedness

The findings indicate that psychological preparedness among young children in volcanic small-island settings is closely shaped by relationships within the *tripusat pendidikan* system of family, school and community. Preparedness did not appear to develop solely as an individual capacity, but through emotional support, everyday interactions and children’s relationships with significant adults. Participants frequently emphasised the importance of calm communication, emotional reassurance and guided coping practices in helping children deal with fear and uncertainty during eruption situations. These interactions reflect processes of co-regulation and emotional scaffolding, where adults help children understand danger, regulate emotions and maintain a sense of safety during crises (Bowlby 1979; De Castro & Pereira 2019). In this setting, teachers, parents and community members functioned not only as sources of disaster-related information but also as important emotional support systems for children.

The findings also showed uneven preparedness across the *tripusat pendidikan* system. Although participants generally demonstrated strong individual coping capacities, communication and institutional coordination remained inconsistent. Limited collaboration, irregular preparedness activities and fragmented institutional support suggest that preparedness within the community relied more on informal caregiving practices and collective support than on structured institutional systems. From a socio-ecological perspective, tripusat pendidikan can therefore be understood as a culturally grounded support system through which children’s psychological preparedness develops through co-regulation, emotional support and shared caregiving practices. Beyond relational support within the *tripusat pendidikan* system, preparedness was also shaped by broader cultural meanings and place-based experiences associated with living in a volcanic environment.

### Cultural and place-based dimensions of preparedness

Psychological preparedness in volcanic small-island communities appeared to be closely connected to cultural values, spirituality, collective memory and everyday experiences of living with environmental risk. Participants described local cultural expressions such as ‘*bakujaga torang pe ana*’, traditional beliefs related to natural signs (*kose*) and collective practices like ‘*kololie kie*’ as important parts of community-based preparedness. These cultural practices shaped how disaster risks were understood, communicated and emotionally managed within the community.

Spiritual and cultural practices also functioned as important psychological resources during disaster situations (Mercer et al. 2012; Rahiem & Rahim 2020). Participants often interpreted volcanic events through religious and collective cultural meanings that helped maintain emotional stability and social cohesion during periods of uncertainty. In this context, preparedness extended beyond technical disaster knowledge and included culturally grounded understandings of danger, safety and collective responsibility.

Place-based experiences further shaped preparedness within the Ternate community. Repeated exposure to Mount Gamalama’s volcanic activity appeared to strengthen adaptive coping practices, community solidarity and emotional attachment to place. These findings highlight the importance of culturally grounded and place-based approaches to disaster preparedness in small-island and hazard-prone settings. These relational and cultural dimensions of preparedness have important implications for the development of child-centred disaster preparedness programmes in hazard-prone communities.

### Implications for child-centred disaster preparedness

The findings highlight several implications for child-centred disaster preparedness in volcanic small-island settings. Disaster risk reduction programmes for young children should incorporate not only physical safety procedures but also psychological and relational aspects of preparedness. Emotional support, co-regulation and developmentally appropriate communication appeared important in helping children cope with fear and uncertainty during disasters. The findings also emphasise the importance of interventions that involve family, school and community as interconnected support systems within the *tripusat pendidikan* framework. In addition, psychosocial support activities, including emotional regulation practices, visual learning media and child-friendly simulations, may help strengthen children’s adaptive coping capacities in disaster situations. Practically, disaster preparedness programmes may benefit from more culturally grounded and child-centred approaches that integrate local values, collective caregiving and community participation.

### Limitations

Several methodological and contextual limitations should be considered when interpreting these findings. The predominance of female participants may have reduced the diversity of perspectives represented, particularly regarding caregiving practices and preparedness experiences among male caregivers. In addition, the study was conducted in Ternate Island, a volcanic small-island setting with distinct socio-cultural and environmental characteristics, which may limit the transferability of the findings to other disaster contexts. The relatively small qualitative sample may also have constrained the range of preparedness experiences captured in the study. Another limitation concerns the use of the PREPARED tool, which was developed within a broader Indonesian population context. Although the instrument demonstrated strong reliability, it may not fully capture the cultural specificity of the Ternate, Gamalama community, including local meanings, spiritual beliefs and indigenous preparedness practices related to volcanic hazards. Future research may therefore benefit from developing more context-sensitive assessment approaches for volcanic small-island communities. The qualitative phase also involved only participants with low to moderate PREPARED scores. Consequently, the experiences of highly prepared stakeholders were not represented in the FGDs, which may have placed greater emphasis on preparedness challenges and vulnerabilities than on adaptive practices among highly prepared individuals. In addition, because the study used a cross-sectional design, it was not possible to examine long-term changes in psychological preparedness, relational dynamics or institutional adaptation over time. Future studies would benefit from longitudinal approaches and more diverse participant backgrounds to better capture the complexity of children’s socio-ecological preparedness systems across disaster contexts.

## Conclusion

Psychological preparedness among ECE stakeholders in Ternate appeared relatively strong at the individual level, particularly in terms of self-efficacy, adaptive coping and general disaster awareness. In contrast, relational and institutional aspects of preparedness, such as coordinated communication, collaborative planning and structured disaster response systems, were less consistently developed. Limited dissemination of preparedness practices, unequal access to training opportunities and the absence of routine simulation exercises further reflected gaps in institutional preparedness. These findings highlight the importance of multi-level preparedness efforts involving families, schools and communities within the *tripusat pendidikan* system. Collaborative disaster workshops, community-based simulations and stronger partnerships with local emergency services may help strengthen social cohesion and collective responsibility during disaster situations. The findings also suggest the importance of integrating psychosocial support, emotional regulation strategies and culturally grounded practices, including storytelling and collective caregiving, into disaster preparedness programmes for young children living in volcanic hazard settings.
